# Differential Cell Metabolic Pathways in Gills and Liver of Fish (White Seabream *Diplodus sargus*) Coping with Dietary Methylmercury Exposure

**DOI:** 10.3390/toxics11020181

**Published:** 2023-02-16

**Authors:** Giuseppe De Marco, Barbara Billè, Fátima Brandão, Mariachiara Galati, Patrícia Pereira, Tiziana Cappello, Mário Pacheco

**Affiliations:** 1 Department of Chemical, Biological, Pharmaceutical and Environmental Sciences, University of Messina, 98166 Messina, Italy; 2 Centre for Environmental and Marine Studies (CESAM) and Department of Biology, University of Aveiro, 3810-193 Aveiro, Portugal

**Keywords:** organic mercury, dietary exposure, white seabream, gills, liver, total Hg accumulation, NMR-based metabolomics, fish metabolome, polar metabolites

## Abstract

Mercury (Hg) is a dangerous and persistent trace element. Its organic and highly toxic form, methylmercury (MeHg), easily crosses biological membranes and accumulates in biota. Nevertheless, understanding the mechanisms of dietary MeHg toxicity in fish remains a challenge. A time-course experiment was conducted with juvenile white seabreams, *Diplodus sargus* (Linnaeus, 1758), exposed to realistic levels of MeHg in feed (8.7 μg g^−1^, dry weight), comprising exposure (E; 7 and 14 days) and post-exposure (PE; 28 days) periods. Total Hg levels increased with time in gills and liver during E and decreased significantly in PE (though levels of control fish were reached only for gills), with liver exhibiting higher levels (2.7 times) than gills. Nuclear magnetic resonance (NMR)-based metabolomics revealed multiple and often differential metabolic changes between fish organs. Gills exhibited protein catabolism, disturbances in cholinergic neurotransmission, and changes in osmoregulation and lipid and energy metabolism. However, dietary MeHg exposure provoked altered protein metabolism in the liver with decreased amino acids, likely for activation of defensive strategies. PE allowed for the partial recovery of both organs, even if with occurrence of oxidative stress and changes of energy metabolism. Overall, these findings support organ-specific responses according to their sensitivity to Hg exposure, pointing out that indications obtained in biomonitoring studies may depend also on the selected organ.

## 1. Introduction

Mercury (Hg) is recognized as a dangerous metallic pollutant because of its persistence and high toxicity to organisms [1], and in recent years its increase in aquatic environments rekindled serious environmental and human health concerns [2]. The high affinity of Hg for cell membrane lipids [3] and its ability to interfere with cellular events such as growth, proliferation, differentiation, or damage repair processes [4] classify it as one of the most hazardous and toxic pollutants. Its presence in aquatic ecosystems is enhanced by natural events such as forest fires and geological emissions, as well as by emissions related to fossil fuel combustion, industrial applications, or mining [5,6,7].

A major concern is the ability of Hg to be easily transferred along trophic chains through bioaccumulation and biomagnification processes and then be accumulated in wildlife at high trophic levels [8,9]. In particular, in aquatic environments, its organic and highly toxic form, methylmercury (MeHg), is produced both in the water column and in sediments by certain sulfate-reducing bacteria and tends to be accumulated mainly in the tissues of aquatic organisms such as fish [10,11]. It is noteworthy that this trace element can be absorbed by aquatic organisms either aqueously, through, e.g., respiratory surfaces, or through the diet, resulting in absorption in the gastrointestinal tract [12,13]. Indeed, due to its high solubility in lipids, single valence, and small size, MeHg can easily cross biological membranes and be completely absorbed from the digestive tract [14] and readily reach the bloodstream for distribution to various organs. Furthermore, it is also known that the elimination rates of the organic form of Hg are very slow, leading to its bioconcentration within the cells of organisms, thus causing biological impairments even at very low doses [15,16].

Although Hg toxicity has been the topic of several studies [17,18,19], clarifying the mechanisms of dietary MeHg toxicity in fish remains challenging. Fish have been widely used in ecotoxicological studies as worthy sentinel organisms [6,20,21,22,23,24,25,26] due to their ability to accumulate and metabolize different contaminants in their tissues and to produce valid and measurable responses (biomarkers) when exposed to stressful conditions, such as the presence of Hg. 

The white seabream, *Diplodus sargus* (Linnaeus, 1758), is a common demersal fish that inhabits infralittoral and circalittoral rocky habitats in the Mediterranean Sea and eastern Atlantic Ocean [27], and it has a relatively long-life span and an omnivorous benthic diet. *D. sargus* accumulates dangerous elements in its tissues, such as Hg [28], even if it is a medium-size fish. These remarkable features make *D. sargus* a valuable bioindicator species for monitoring aquatic contaminants. In previous studies [29,30], white seabream was used as animal model to shed light on MeHg neurotoxicity after dietary exposure by combining bioaccumulation levels, oxidative stress profiles, and behavior assessment. However, these conventional biochemical assays often need the support of high-throughput methods to gain more comprehensive insights into the biological effects induced by environmental pollutants on target organisms. Environmental metabolomics is regarded as a very accurate method of investigation that assesses variation in the physiological state of organisms coping with different environmental scenarios, even under experimental conditions, through the simultaneous evaluation of a large number of biomolecules [31]. Lin et al. [32] identified metabolomics as the comprehensive analysis of all low-molecular-weight (<1500 Da) endogenous metabolites that may vary according to the physiological state, developmental stage, or pathological state of cells, tissues, or organs, or of the entire organism under investigation. In particular, metabolomics based on proton nuclear magnetic resonance (^1^H NMR) allows for the simultaneous analysis of a wide range of metabolites involved in different metabolic pathways, thus offering numerous advantages for elucidating organism–environment interactions. In addition, it allows for the identification of novel metabolic biomarkers in response to stress in organisms provoked by changes in abiotic factors, diseases, or environmental pollutants [31,32,33,34,35,36,37,38,39,40,41]. 

Although previous studies have highlighted the detrimental impact of MeHg on the brain of white seabream [29,30], so far, to our knowledge, no studies have examined its effects on organs such as gills and liver through the use of environmental metabolomics. Due to certain features (i.e., large surface area in close contact with dissolved water pollutants, intense blood flow, and high cell regeneration, which are useful to indicate more recent pollutant exposures), fish gills have been considered an important target of aquatic pollution, and therefore they are frequently used in environmental biomonitoring studies [40,41,42]. Furthermore, gills are also capable of storing pollutants absorbed in the gut [43], including MeHg [17,44]. Similarly, the liver is widely used to assess the health status of fish [41,45], as it is an important organ of metabolic activity including detoxification processes. Furthermore, its important role in bioaccumulation, transformation, and Hg cycling processes confirms the importance of liver for understanding the toxic effects and metabolic malfunctions induced by possible contact with this pollutant [17,20,44]. 

In order to further improve the current knowledge on the effects of Hg on aquatic biota, the present study was designed to compare the cytotoxic effects of dietary MeHg on gills and liver of white seabream, *D. sargus*, through the evaluation of Hg bioaccumulation and metabolomic profiling. In detail, fish were exposed by diet to a realistic concentration of MeHg (8.7 μg g^−1^ dry feed) over 14 days. Successively, a post-exposure period of 28 days was also taken into consideration in order to estimate the recovery ability of the two selected target organs in fish. 

## 2. Materials and Methods

### 2.1. Chemicals

Deuterium oxide (D_2_O, heavy water; 99.8 atom %D; CAS-No. 7789-20-0) for NMR spectroscopy was obtained from Armar AG Chemicals (Dottingen, Switzerland). The 2,2-dimethyl-2-silapentane-5-sulfonate sodium salt (DSS; 97%, molecular weight: 218.32 g mol^−1^; CAS-No. 2039-96-5) was purchased from Sigma-Aldrich (Milan, Italy). The other chemicals required to conduct the metabolomics analysis, namely, methanol (molecular weight: 32.04 g mol^−1^; CAS-No. 67-56-1) and chloroform (molecular weight: 119.38 g mol^−1^; CAS-No. 67-66-3) were also purchased from Sigma-Aldrich (Milan, Italy). For the experimental exposure, MeHg chloride (molecular weight: 251.08 g mol^−1^; CAS-No. 115-09-3) was purchased from Sigma-Aldrich Chemical (Madrid, Spain). Other routine chemicals used in this work were of analytical grade and acquired from local suppliers.

### 2.2. Experimental Set-Up and Sampling

Juvenile white seabreams (*Diplodus sargus*), provided by the Aquaculture Research Station (IPMA—Olhão, Portugal), were used in the experiment. Fish wellbeing deserved permanent attention in accordance with national and international guidelines for the protection of animal welfare. Fish were allowed to acclimatize to experimental conditions and routines for two weeks prior to MeHg exposure. Water temperature, salinity, and pH were monitored daily, varying as follows, respectively: 13.5 ± 0.3 °C, 35 ± 2, and 7–8. 

Fish were held in 300 L fiberglass tanks in an average density of 0.056 kg L^−1^ (initial fish weight: 124 ± 11 g; initial total length: 18 ± 0.6 cm) under a 10 h light:14 h dark photoperiod. Seawater was renewed daily (around 80%), and fish were fed once a day, namely, 1–2 h before water renewal. In sampling days, fish were not fed in the 12 h preceding handling that started around 09:00 am. 

Fish were fed with feed (3 mm pellets) produced by SPAROS Company (Olhão, Portugal) composed by 44% protein and 16% lipids. A solution of MeHg chloride (prepared in ethanol) was added during the process of pellet production, with a homogenous distribution of the toxicant throughout the batch. MeHg levels in contaminated pellets (8.7 ± 0.5 μg g^−1^ dry weight) used to expose fish to this Hg form were similar to those previously detected in natural food of *D. sargus* (e.g., *Nereis diversicolor*), as previously described [29]. Control fish were fed with uncontaminated pellets, with the same size and nutritional formulation, which were produced following an identical methodology but without the addition of MeHg (residues lower than 0.01 μg g^−1^).

**Figure 1:**
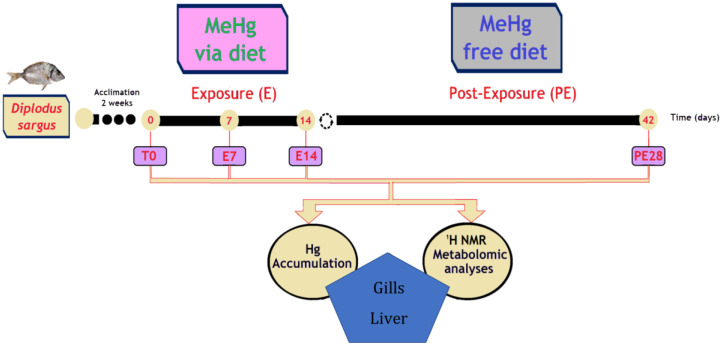
Experimental design with white seabream, *Diplodus sargus*, comprising an acclimation time of 2 weeks (T0) followed by an exposure period to MeHg via diet for 7 (E7) and 14 days (E14). Thereafter, fish were fed with a MeHg-free diet for 28 days (Day 42; PE28) to allow recovery. In parallel, control groups of fish were also considered. At each experimental time, gills and liver were collected to measure levels of Hg and to perform a metabolomic analysis.

Fish were exposed to MeHg for 7 (E7) and 14 (E14) days. Then, to potentially allow recovery for 28 days, fish started to be fed with uncontaminated pellets (post-exposure; PE28) (Figure 1). Thus, the experiment had a total duration of 42 days. At each sampling time (E7, E14, and PE28), fish were sacrificed for two different analyses as follows: (i) eight fish for total Hg determinations; and (ii) six fish for metabolomics analysis (Figure 1). During sampling, fish were anesthetized with 0.2 mg L^−1^ tricaine methanesulfonate (MS-222; molecular weight: 261.29 g mol^−1^; CAS-No. 886-86-2) purchased from Sigma-Aldrich, China, and buffered with NaHCO_3_. Fish were then weighed, measured, sacrificed by cervical transection, and properly bled from the cardinal vein (using heparinized Pasteur pipettes). Gills and liver were excised, and the two sets of samples stored at −80 °C until further processing for Hg determination and metabolomics.

### 2.3. Total Hg Determination

Firstly, gills and liver samples were lyophilized, homogenized, and then analyzed for total Hg (tHg) in an advanced mercury analyzer (AMA) (AMA254, LECO Instruments), according to the U.S. EPA method 7473 [46]. Briefly, the initial preparation step of the analytical process of AMA was removing the moisture through a drying process to concentrate Hg in the sample. Thermal decomposition (around 750 °C) was used to pyrolytically reduce Hg in the sample to its elemental form. Elemental Hg was then trapped on a gold amalgamator and eventually liberated by heating the amalgamator. Elemental Hg was transported by a stream of oxygen and measured by atomic absorption spectrometry [47]. Certified reference materials (fish protein: DORM-4; dogfish liver: DOLT-4) from the Canadian National Research Council were used to ensure the accuracy of the procedure, and the obtained values were consistent with the certified ones.

### 2.4. Metabolomics Analysis

#### 2.4.1. Extraction of Metabolites

Polar metabolites were extracted from liver and gill tissues of white seabreams (n = 6 per condition at each sampling time) by applying a “two-step” methanol/chloroform/water protocol, as reported in detail in previous works [35,40]. Briefly, frozen 100 mg sub-samples of each fish tissue were transferred in 2 mL Eppendorf tubes with the addition of cold methanol (4 mL g^−1^) and cold distilled water (0.85 mL g^−1^) in order to be homogenized using a TissueLyser LT bead mill (Qiagen, Hilden, Germany), with the inclusion of a 3.2 mm stainless steel bead in each tube. Therefore, homogenization took place for 10 min at 50 vibrations/s. The homogenates, after addition of chloroform (4 mL g^−1^) and distilled water (2 mL g^−1^), were then vortexed for 60 s to be mixed and left on ice for 10 min to partition. Afterward, samples were centrifuged for 5 min at 2000 g at 4 °C, resulting in a triphasic mixture. The upper methanol layer (600 µL) with polar metabolites was then carefully removed and transferred into clean vials to be dried in a centrifugal vacuum concentrator (Eppendorf 5301). The resulting pellet was then kept at −80 °C. Prior to NMR analysis, the dried extracts were resuspended in 600 μL of a 0.1 M sodium phosphate buffer (pH 7.0, 10% D_2_O) with 1 mM DSS, used as internal reference, and then transferred to a 5 mm diameter NMR tube. DSS acts as an internal standard and provides a chemical shift reference (δ = 0.0 ppm) for the NMR spectra, while D_2_O provides a deuterium lock for the NMR spectrometer. 

#### 2.4.2. ^1^H NMR-Based Metabolomics and Spectral Pre-Processing

A Varian-500 NMR spectrometer working at a spectral frequency of 499.74 MHz at 298 K was used to analyze fish tissue extracts. To obtain one-dimensional (1-D) ^1^H NMR spectra, a PRESAT pulse sequence was applied for suppressing the resonance of residual water, with 6 kHz spectral width and a 2.0 s relaxation delay. A total of 128 transients was collected into 16,384 data points requiring a 10 min acquisition time for each sample under investigation. All data sets were zero filled to 32,768 data points, and exponential line-broadenings of 0.5 Hz were used before application of the Fourier transformation. A Chenomx Processor, a module of Chenomx NMR Suite (version 5.1; Chenomx Inc., Edmonton, Canada) software, was then used for manually phasing, baseline-correction, and calibration (DSS at 0.0 ppm) of all ^1^H NMR spectra acquired. The Chenomx 500 MHz library database, another module included into the Chenomx NMR Suite software, was utilized for identifying the different peaks profiled within the acquired ^1^H NMR spectra from each seabream gill and liver in order to be assigned with reference to known chemical shifts and peak multiplicities. Furthermore, the use of Chenomx NMR Profiler, another module also included in the Chenomx NMR Suite software, allowed us to quantify the level of each individual metabolite detected in the ^1^H NMR spectra using the known concentration of the internal standard DSS, previously introduced in each sample prior to the NMR analysis [48,49]. Thereafter, the concentration of the metabolites of interest recorded in seabream gills and liver at all the experimental times was expressed as mean ± standard deviation (SD). 

### 2.5. Data Statistical Analysis

GraphPad software (Prism 7.0, San Diego, CA, USA) was used for all the statistical analyses. Bioaccumulation data on the levels of total Hg (μg g^−1^) measured in seabream gills and liver were presented as mean ± SD, and statistically significant differences (*p* < 0.05) between the control and exposed fish at all the experimental times were established by application of a two-way analysis of variance (ANOVA) followed by the post hoc Sidak’s multiple comparisons test. Metabolomics data were expressed in mM as mean ± SD. For each fish organ, metabolite changes were calculated via the ratio between the averages of exposed fish at each sampling time and control peak areas. The metabolic dataset was tested for normality using the Shapiro–Wilk distribution test, and after confirmation of normal distribution, data homogeneity was evaluated through the Levene test. Data were analyzed by one-way ANOVA using Dunnett’s multiple comparison test, which was aimed at finding significant differences between the control and exposed fish at each experimental time. Data were considered statistically significant at *p* < 0.05. 

## 3. Results

### 3.1. Total Hg Accumulation

**Figure 2:**
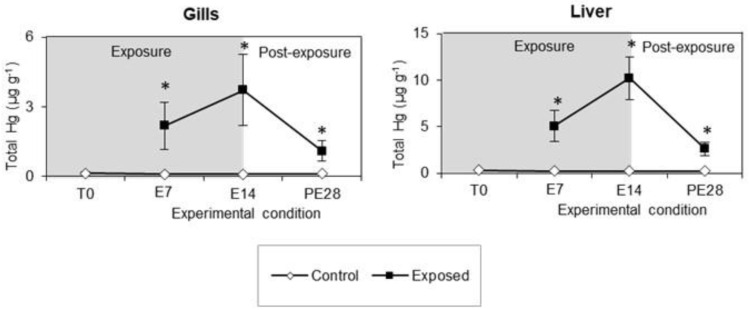
Total Hg level (tHg; μg g^−1^) measured in the gills and liver of white seabream exposed to MeHg and in control fish at each experimental time over 14 days of exposure (7 and 14 days, corresponding to E7 and E14) and 28 days of post-exposure (day 42, corresponding to PE28). Data correspond to mean ± standard deviation (n = 8). Significant differences (Sidak’s test; *p* < 0.05) in relation to the control group are indicated by * for each experimental time.

The levels of total Hg (tHg) measured at all experimental times in the gills and liver of white seabreams exposed to MeHg by diet, as well as those of control fish, are depicted in Figure 2. Overall, a significant rise of tHg levels was observed in both fish organs throughout the 14-day exposure period, followed by a drop during the post-exposure time of 28 days. In particular, the highest tHg levels were detected at E14, both in the gills (3.7 μg g^−1^) and in the liver (10.2 μg g^−1^), with the latter organ depicting levels 2.7 times higher. It is worth noting that during the post-exposure period, tHg levels decreased for both organs but remained above the values recorded in the control fish.

### 3.2. Metabolomics 

#### 3.2.1. ^1^H NMR Spectroscopy of Gill and Liver Extracts of Unexposed Fish

**Figure 3:**
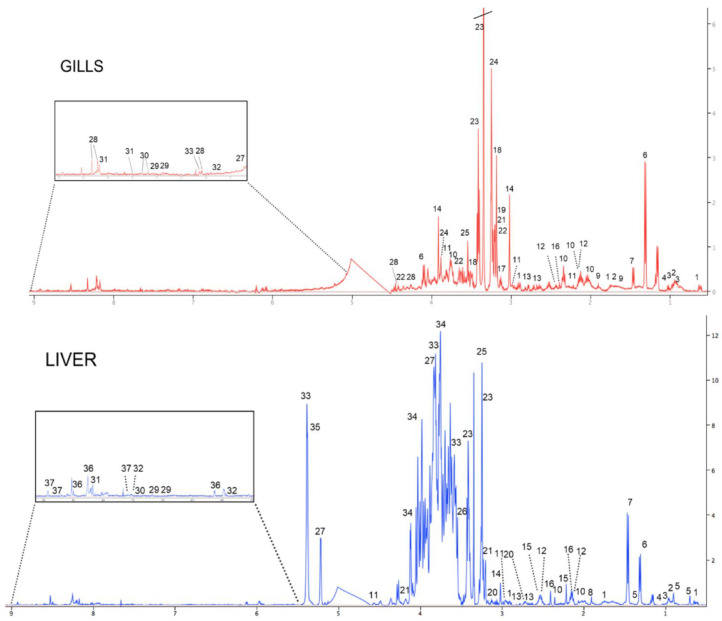
Representative 1-D 500 MHz ^1^H NMR spectra of the gills and liver of white seabream, *Diplodus sargus*, from the control group: (1) DSS, (2) leucine, (3) isoleucine, (4) valine, (5) cholate, (6) lactate, (7) alanine, (8) acetate, (9) arginine, (10) glutamate, (11) glutathione, (12) glutamine, (13) aspartate, (14) creatine, (15) acetone, (16) succinate, (17) malonate, (18) choline, (19) acetylcholine, (20) cystathionine, (21) phosphocholine, (22) glycerophosphocholine, (23) taurine, (24) betaine, (25) TMAO, (26) glycine, (27) glucose, (28) inosine, (29) tyrosine, (30) phenylalanine, (31) hypoxanthine, (32) uracil, (33) maltose, (34) fructose, (35) glycogen, (36) ATP/ADP, and (37) niacinamide.

Typical 1-D ^1^H NMR spectra of the metabolome of the gills and liver of juvenile white seabream *D. sargus* from the control group are shown in Figure 3. Among the several metabolites identified, it was found that gills were dominated by the osmolyte taurine, whose concentration was found to be about eight times higher than that of the other metabolites. Conversely, the metabolome of liver was characterized by a dominant presence of carbohydrates (e.g., glucose, maltose, fructose), whose concentrations were up to 60 times higher than those of the other detected metabolites. Among the other prominent classes of compounds found in the metabolome of both fish organs, amino acids (e.g., glutamate, alanine), Kreb’s cycle intermediates (e.g., succinate), and nucleotides (e.g., uracil) were observed. It is also worth noting that some metabolites such as arginine, malonate, choline, acetylcholine, betaine, and inosine were exclusively detected in the gills, whereas cholate, acetate, acetone, cystathionine, trimethylamine N-oxide (TMAO), maltose, fructose, glycogen, adenosine triphosphate/adenosine diphosphate (ATP/ADP), and niacinamide were observed only in the liver.

#### 3.2.2. Metabolome of Gills in MeHg-Exposed Fish

Dietary exposure to MeHg provoked alterations in different classes of metabolites in fish gills at each experimental time (Table 1). Indeed, a significant increase in the levels of several amino acids (leucine, isoleucine, valine, alanine, tyrosine, phenylalanine) was recorded during the exposure period (E7; E14). However, it is worth noting the significant decline observed in almost all amino acids at the post-exposure time that lasted 28 days (PE28). Regarding energy metabolism, a significant notable rise in lactate was noted at all the experimental times with respect to the control, combined with a complex pattern of variation in the levels of malonate, glycogen, and glucose. Furthermore, significant increases in the levels of osmolytes (taurine, glycerophosphocholine), the neurotransmitter acetylcholine, and miscellaneous metabolites (phosphocholine, inosine) were also observed with respect to the control at all MeHg experimental times and in the post-exposure period, in which fish were fed with a MeHg-free diet.

#### 3.2.3. Metabolome of Liver in MeHg-Exposed Fish

Dietary exposure to MeHg provoked alterations in different classes of metabolites in fish liver at each experimental time (Table 2). In regard to amino acids, a significant increase was found in the level of alanine at all the exposure and post-exposure experimental times combined with a significant drop in the concentration of glutamine and glutamate. In terms of energy production, the concentration of glucose decreased significantly at all the experimental times, whilst the ATP/ADP ratio increased significantly. Overall, with regard to other metabolites, significant reductions in glutathione at PE28 and creatine levels at all the experimental times were detected, associated with a significant enhancement of cystathionine and niacinamide levels at both the exposure and post-exposure experimental times.

## 4. Discussion

To date, the use of metabolomics has proven to be an extremely effective tool for investigations of the perturbed metabolic pathways in aquatic biota triggered by a variety of pollutants [26,31,32,49,50,51], including Hg [20,40,41]. In this work, the metabolomic approach was applied on gills and liver of white seabream, *D. sargus*, with the aim of elucidating the cytotoxic effects of dietary MeHg and the potential recovery ability of the selected target organs during a successive post-exposure period, revealing differential impairments in various metabolic pathways.

The use of ^1^H NMR-based metabolomics allowed for the simultaneous detection of 27 metabolites in the gills and 31 metabolites in the liver. Interestingly, some metabolites were in common among the two fish organs, though organ-specific metabolites were also observed. In detail, the presence of metabolites involved in neurotransmission (i.e., acetylcholine, choline) and osmoregulation (betaine) was observed solely in the gills, whereas some carbohydrates and molecules involved in energy metabolism (i.e., fructose, maltose, glycogen, ATP/ADP, niacinamide), as well as ketone bodies (i.e., acetate, acetone), were predominantly recorded in the liver. The evidence of organ-specific metabolomic profiles, both in terms of presence of specific metabolites and in terms of their concentrations, as previously described in other aquatic organisms [35,52], may be explained by the differential physiological functioning of the two examined organs. In fact, the gills of fish are a multipurpose organ that, in addition to respiratory gas exchange, play prominent roles in feeding by filtering food particles, osmotic regulation, active ion transport, and nitrogenous waste excretion [35,43]. Conversely, the liver of fish is a target organ involved in a number of metabolic activities, including energy and carbohydrate metabolism [35,53], besides for playing a key role in accumulation, biotransformation, and cycling of environmental pollutants, because of its effective detoxification system useful to counteract the harmful effects of hazardous chemicals [54]. 

The different physiological role of the two examined organs would also explain their dissimilar capacity to accumulate Hg. In fact, in the present study it was found that the liver, responsible for detoxification of pollutants, showed more than two times higher levels of total Hg than the gills following MeHg dietary exposure. In both organs, total Hg levels tended to decrease during the post-exposure period, though concentrations did not reach baseline levels after 28 days of depuration, as they remained statistically higher than the values recorded in the control, thereby attesting to the strong persistence of Hg within biological tissues [29,55,56,57]. Comparable results were also observed in white seabream following waterborne exposure and post-exposure periods to inorganic mercury (iHg) in a study aimed at elucidating Hg toxicokinetics [58]. Overall, the differential bioaccumulation of Hg, together with the physiological specificities of the two examined fish organs, were on the basis of organ-specific impairments observed in a variety of metabolic pathways. 

### 4.1. MeHg-Induced Metabolome Changes in Fish Gills

It is well known that free amino acids are the most abundant components in the gills of fish [38,41]. In this study, the dietary exposure of white seabreams to realistic concentration of MeHg (8.7 μg g^−1^) provoked, at all the selected exposure times (E7, E14), an increase in the level of branched-chain amino acids (BCAAs, including leucine, isoleucine, and valine), tyrosine, phenylalanine, and alanine, likely due to a MeHg-induced protein catabolism, followed by a noteworthy drop in their concentrations at the successive post-exposure period (PE28), perhaps associated with a possible occurrence of protein biosynthesis as a repair or cell renewal/turnover process. As is widely documented in the literature, BCAAs play a key function in the immune system by both regulating protein turnover and being precursors for the biosynthesis of new molecules (i.e., immunoglobulins, interleukins, and chemokines), which are essential for cells such as lymphocytes to correctly perform their functions [59]. Thus, the rise in BCAA levels could suggest the involvement of the immune system to counteract the detrimental impact of MeHg in the gills. The occurrence of intense protein turnover activity appears also to be confirmed by the increased levels of alanine during the exposure period. Indeed, this amino acid is implicated in nitrogen metabolism, which underlies the processes of nitrogen waste excretion occurring in the gills as a consequence of protein catabolism [43,60]. Therefore, these results hint at the existence of ammonia excretion impairment triggered by dietary exposure to MeHg, which nevertheless seemed to be slightly mitigated during the post-exposure period. 

Moreover, the higher levels of tyrosine and phenylalanine, coupled with the enhancement of acetylcholine detected at all the experimental times, may be associated with alterations in the neurotransmission system. As a matter of fact, tyrosine and phenylalanine are precursors of the neurotransmitter dopamine, which is implicated in the dopaminergic system [61], while acetylcholine is the major neurotransmitter involved in the cholinergic system. It is known that both these nervous systems are strictly associated with the hypoxic signaling of fish gills [62]. Hence, the changes observed in these metabolites may be related to the disruption of the gas exchange processes [63], therefore corroborating the MeHg neurotoxic effects already documented in previous works carried out on the brain of white seabreams upon exposure to Hg forms [29,39]. Moreover, a plausible reason for the increase in acetylcholine could also be the inhibition of acetylcholine esterase (AChE) activity, already demonstrated in fish exposed to MeHg [64,65]. 

The depression of neuronal activity could therefore result in an alteration of the normal functioning of the gills, also supported by the increased concentration of alanine as discussed above. The increased energy demand would seem to be partly counterbalanced by the enhanced levels of lactate, which may suggest a shift towards anaerobic energy metabolism [41]. Furthermore, the increased levels of malonate, a precursor for fatty acid synthesis [66], indicate also impairments in the energy metabolism related to the highly biosynthetic activity aimed at mitigating the harmful impact caused by dietary MeHg exposure in white seabream. 

Besides changes in energy metabolism, in the gills of fish the osmoregulation system appeared also to be disrupted following exposure to MeHg via diet, as supported by the increased levels of taurine and glycerophosphocholine observed during all the exposure and post-exposure times. The MeHg effect on the concentration of osmolytes confirms the high sensitivity of the osmoregulatory system in aquatic organisms exposed to Hg species and other classes of pollutants [20,67,68]. However, as already reported in previous works [20,35], it is noteworthy to highlight that the raised levels of glycerophosphocholine and phosphocholine, both precursors of phosphatidylcholine, could suggest the occurrence of phospholipid breakdown, leading to cell membrane instability, persisting even during the following 28 days when the specimens were fed by a MeHg-free diet [69]. 

Moreover, among the changes observed in the metabolome of fish gills, it is interesting to note the increased levels of inosine, likely due to the breakdown of adenosine, which are a sign of cellular stress, commonly occurring during high energy demand [70]. In addition, Li et al. [71] pointed out how inosine may exert a vasodilatory effect on the gill lamellae of fish. Therefore, the increase in this metabolite, persisting even during the post-exposure period, hints at the emergence of an energy deficit in the branchial tissue, probably due to the intense biosynthesis activity elicited by dietary MeHg exposure.

Finally, it is interesting to note how certain metabolites, and in particular those involved in neurotransmission, osmoregulation, and cell membrane stability, appeared to be altered even during the post-exposure period (PE28). This may reflect a higher sensitivity of certain metabolic pathways than others to MeHg exposure and emphasize the persistence of certain harmful effects triggered by MeHg, even after a considerable time beyond the exposure phase [29,72].

### 4.2. MeHg-Induced Metabolome Changes in Fish Liver

The MeHg dietary exposure of white seabreams provoked alterations in some metabolic pathways also at the hepatic level that, with some exceptions, appeared differently from those revealed at the gills. Among amino acids, the changes observed in the levels of alanine were comparable with those recorded in the gills. In fact, the concentrations of alanine increased during all the exposure and post-exposure times, thus confirming the impact of MeHg in nitrogen metabolism [43,64]. 

Contrarily to the gills, changes in the amino acids glutamate and glutamine were observed in the liver. These metabolites, which are precursors of glutathione, were found to be reduced especially during the selected exposure times. These impairments, taken together with the drop recorded in the levels of glutathione, support the involvement of the antioxidant system to face the impact triggered by MeHg exposure, as already widely reported in the literature, since it is known that a decisive way to initiate Hg toxicity in organisms is through oxidative stress [20,29,35,40,41]. Moreover, the decrease in glutathione levels could also be associated with the high MeHg reactivity towards the reduced sulphydryl group [73]. In this regard, Akiyama et al. [74] emphasized the relevance of the enzyme cystathionine γ-lyase (CSE) in producing reactive sulphur species useful in mitigating the detrimental impact of MeHg. Therefore, the enhancement of cystathionine, the main substrate of CSE, detected in the liver of fish by the metabolomics approach, could suggest the MeHg-induced inhibition of CSE. 

Moreover, in the liver of seabreams dietarily exposed to MeHg, variations in the levels of glucose, ATP/ADP, creatine, and niacinamide were also noted and could be ascribed to disruptions in energy metabolism. In fact, the drop revealed in the glucose levels and the unchanged level of glycogen, despite the regular food administration to seabreams, suggest hepatic gluconeogenesis inhibition, contrarily to what was observed in the golden grey mullet collected in a highly Hg-polluted environment [20,41]. However, the increased ATP/ADP levels, coupled with increased concentrations of niacinamide, which is the NAD precursor, could indicate high energy production in the liver necessary to sustain the detoxification and repairing actions [37] to face the detrimental effects of MeHg. Furthermore, creatine is a key molecule in the transfer of energy through organs and tissues [75]. Thus, its reduction in the fish liver, despite the high ATP/ADP levels detected, could be justified by its transfer to tissues with a higher energy demand [76,77].

Contrary to the results observed in the gills, the levels of several metabolites detected at the hepatic level appeared to be altered even at PE28, indicating a higher sensitivity to MeHg of this organ than the gills, probably linked also to the higher Hg accumulation recorded in the liver. Therefore, these findings further support the occurrence of organ-specific responses elicited by fish in accordance with their sensitivity to Hg exposure, since it has been previously documented that MeHg is able to affect differently even parts of the same organ, as observed within the brain optic tectum of white seabreams [39]. 

## 5. Conclusions

In this work, the NMR-based metabolomic approach allowed us to shed light into the toxic metabolic pathways triggered by dietary MeHg exposure in juveniles of white seabream, *Diplodus sargus*, as well as to reveal organ-specific cytotoxicity mechanisms induced by MeHg in the gills and liver. In brief, following exposure to MeHg via diet, an intense energy catabolism associated with disruption of neurotransmission and osmoregulation systems was observed in the gills, whereas impaired antioxidant and detoxification systems were revealed in the liver, coupled with changes in the energy metabolism. Although the post-exposure period of 28 days had allowed a partial recovery of both target organs, there was still evidences of oxidative stress and changes of the energy metabolism, revealing long-term effects of dietary MeHg in fish. Overall, organ-specific cytotoxicity mechanisms of dietary MeHg exposure were discerned, pointing out the vulnerability of fish health to this Hg form at highly impacted ecosystems and the need for increased surveillance in this direction, therefore emphasizing the urgency of the further development of new remediation strategies against this persistent environmental pollutant.

## Figures and Tables

**Table 1:** Percent changes in concentrations of metabolites in the gills of white seabream (*D. sargus*) following MeHg-dietary exposure (E7, E14) and post-exposure (PE28) periods, in relation to control groups (n = 6) (Dunnett’s test, * *p* < 0.05; ↑ and ↓ indicate an increase and decrease in respect to the control group, respectively).

Metabolites	Gills		
	E7	E14	PE28
***Amino acids***			
Leucine	↑ 12%	↑ 59% *	↓ 12%
Isoleucine	↑ 10%	↑ 37% *	↓ 21% *
Valine	↑ 20% *	↑ 43% *	↓ 16%
Alanine	↑ 31% *	↑ 65% *	↓ 4%
Tyrosine	↑ 9%	↑ 27% *	↓ 37% *
Phenylalanine	↑ 34% *	↑ 54% *	↓ 24% *
***Energy-related***			
Lactate	↑ 102% *	↑ 166% *	↑ 99% *
Malonate	↑ 32% *	↑ 64% *	↓ 16% *
Glycogen	↓ 69% *	↓ 31% *	↑ 5%
Glucose	↓ 18% *	↑ 17% *	no change
***Osmolytes***			
Taurine	↑ 58% *	↑ 64% *	↑ 156% *
Glycerophosphocholine	↑ 122% *	↑ 162% *	↑ 228% *
***Neutransmitters***			
Acetylcholine	↑ 68% *	↑ 50% *	↑ 149% *
***Miscellaneous***			
Phosphocholine	↑ 83% *	↑ 126% *	↑ 116% *
Inosine	↑ 67% *	↑ 195% *	↑ 111% *

**Table 2:** Percent changes in concentrations of metabolites in the liver of white seabream (*D. sargus*) following MeHg-dietary exposure (E7, E14) and post-exposure (PE28) periods in relation to control groups (n = 6) (Dunnett’s test, * *p* < 0.05; ↑ and ↓ indicate an increase and decrease in respect to the control group, respectively).

Metabolites	Liver		
	E7	E14	PE28
***Amino acids***			
Alanine	↑ 32% *	↑ 30% *	↑ 13%
Glutamate	↓ 32% *	↓ 73% *	↓ 42% *
Glutamine	↓ 33% *	↓ 24% *	↓ 7%
***Energy-related***			
Glucose	↓ 41% *	↓ 52% *	↓ 41% *
ATP/ADP	↑ 18%	↑ 40% *	↑ 31% *
Glucose	↓ 41% *	↓ 52% *	↓ 41% *
ATP/ADP	↑ 18%	↑ 40% *	↑ 31% *
***Miscellaneous***			
Creatine	↓ 20% *	↓ 39% *	↓ 27% *
Cystathionine	↑ 2%	↑ 79% *	↑ 112% *
Glutathione	↑ 6%	↓ 4%	↓ 16% *
Niacinamide	↑ 55% *	↑ 58% *	↑ 73% *

## Data Availability

The data presented in this study are available upon request from the corresponding author.
